# Utilizing a Spiro Core with Acridine- and Phenothiazine-Based New Hole Transporting Materials for Highly Efficient Green Phosphorescent Organic Light-Emitting Diodes

**DOI:** 10.3390/molecules23040713

**Published:** 2018-03-21

**Authors:** Ramanaskanda Braveenth, Il-Ji Bae, Ji-Hun Han, Wu Qiong, Guk Seon, Kanthasamy Raagulan, Kihun Yang, Young Hee Park, Miyoung Kim, Kyu Yun Chai

**Affiliations:** 1 Division of Bio-Nanochemistry, College of Natural Sciences, Wonkwang University, Iksan City, Chonbuk 570-749, Korea; braveenth.czbt@gmail.com (R.B.); wlgns3931@naver.com (J.-H.H.); wuqiong9308@126.com (W.Q.); guksun92@daum.net (G.S.); raagulan@live.com (K.R.); kihun0432@wku.ac.kr (K.Y.); dudgml9192@naver.com (Y.H.P.); 2 Nano-Convergence Research Center, Korea Electronics Technology Institute, Jeonju 54853, Korea; ijbae@keti.re.kr

**Keywords:** organic light-emitting diodes, spirobifluorene, hole transporting materials, green phosphorescence, phenothiazine, acridine

## Abstract

Two new hole transporting materials, 2,7-bis(9,9-diphenylacridin-10(9*H*)-yl)-9,9′ spirobi[fluorene] (SP1) and 2,7-di(10*H*-phenothiazin-10-yl)-9,9′-spirobi[fluorene] (SP2), were designed and synthesized by using the Buchwald–Hartwig coupling reaction with a high yield percentage of over 84%. Both of the materials exhibited high glass transition temperatures of over 150 °C. In order to understand the device performances, we have fabricated green phosphorescent organic light-emitting diodes (PhOLEDs) with SP1 and SP2 as hole transporting materials. Both of the materials revealed improved device properties, in particular, the SP2-based device showed excellent power (34.47 lm/W) and current (38.41 cd/A) efficiencies when compare with the 4,4′-bis(*N*-phenyl-1-naphthylamino)biphenyl (NPB)-based reference device (30.33 lm/W and 32.83 cd/A). The external quantum efficiency (EQE) of SP2 was 13.43%, which was higher than SP1 (13.27%) and the reference material (11.45%) with a similar device structure. The SP2 hole transporting material provides an effective charge transporting path from anode to emission layer, which is explained by the device efficiencies.

## 1. Introduction

Organic light-emitting diodes (OLEDs) are important because of their valuable applications in flat panel displays and research studies. In the past decade, enormous developments have been seen in both the fabrication technique of and material developments in OLEDs. The improvement of hole transporting materials (HTMs) could enhance device performance, which promotes hole transportation and the injection mechanism from anode to emissive layer (EML). Long-operation and stable OLEDs require highly efficient hole transporting materials with higher glass transition temperatures of over 100 °C [1,2,3,4,5,6,7,8]. The most frequently used HTMs are 4,4′-bis(*N*-phenyl-1-naphthylamino)biphenyl (NPB), 4,4′,4″-tris(*N*-carbazolyl)-triphenylamine (TCTA), 4,4′-bis(3-methylphenylphenylamino)biphenyl (TPD), and di-[4-(*N*,*N*-ditolyl-amino)phenyl]cyclohexane (TAPC) for their prominent hole transporting capabilities and suitable frontier molecular orbital (FMOs) energies. Unfortunately, these materials do not provide sufficient morphological stability during device operation and device construction processes, which lead directly to energy loss and lack of efficiencies with the degradation of the devices. This occurs because of the lower glass transition temperature of frequently used hole transporting materials, such as NPB and TPD, which are 98 °C and 65 °C for, respectively [9,10,11,12,13,14,15].

Many core structures have been developed and modified to overcome these reported issues, notably thermal stabilities [15]. The most ordinarily hole transporting core structures are arylamine, carbazole, and spiro-linked amines. spirobifluorene was one of the key molecules to achieve high efficiencies with stable morphological properties through modification at both the second and seventh positions by incorporating different electron-donating derivatives. Moreover, spiro molecules provide a rigid core along with a twisted structure at the ninth position of the fluorene unit [16,17,18,19,20,21,22,23,24,25]. Spiro-annulated molecules are connected with two conjugated moieties by a tetrahedral bonding atom, which minimizes the close stacking of molecules and enhances the rigidity of the core [26]. Recently, our group reported that spirobifluorene core-based hole transporting materials with higher thermal stabilities exhibited similar efficiencies when compared with NPB HTM for red phosphorescent organic light-emitting diode (PhOLED) applications. However, we did not achieve enough efficiencies to recommend replacing the widely used NPB hole transporting material [27].

In this study, we have further modified the central spirobifluorene by attaching diphenylacridine (SP1) and phenothiazine (SP2) donor moities at both the second and seventh positions. Both of the synthesized materials revealed high glass transition temperatures of over 150 °C, which were applied on green phosphorescent OLEDs to investigate the efficiency of improvements. The phenothiazine (SP2)-based material showed excellent quantum efficiency of 13.43%, which was higher than the reference NPB-based (11.45%) reference device.

## 2. Results and Discussion

### 2.1. Synthesis

**Scheme 1:**
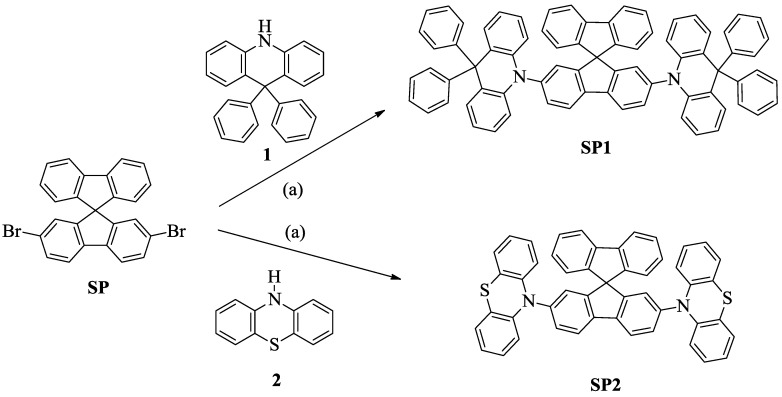
Synthesis of diphenylacridine (SP1) and phenothiazine (SP2). Reagents and reaction condition: (a) Pd(OAc)_2_, NaO*t*Bu, 10% *t*-Bu_3_P in toluene, anhydrous toluene, and anhydrous tetrahydrofuran (THF) at 110 °C SP- spiro.

Our designed molecules, SP1 and SP2, were synthesized by using the Buchwald–Hartwig coupling reaction with excellent yields of 84% and 92% (Scheme 1).

The coupling reaction was held between 2,7-dibromo-9,9′-spirobi[fluorene] (SP), 9,9-diphenyl-9,10 dihydroacridine (**1**), and 10*H*-phenothiazine (**2**). The reaction used palladium acetate (Pd(OAc)_2_) as catalyst along with a tert-tributylphosphine (*t*-Bu_3_P) ligand, and a sodium tert-butoxide (NaO*t*Bu) base. The target molecules of pure SP1 were achieved by using silica column chromatography, while SP2 was purified by a recrystallization technique. Both of the materials were subjected to NMR (^1^H, ^13^C) spectroscopy and mass spectrometry analysis after the purification process to confirm the expected target molecules.

### 2.2. Thermal and Morphological Properties

**Figure 1:**
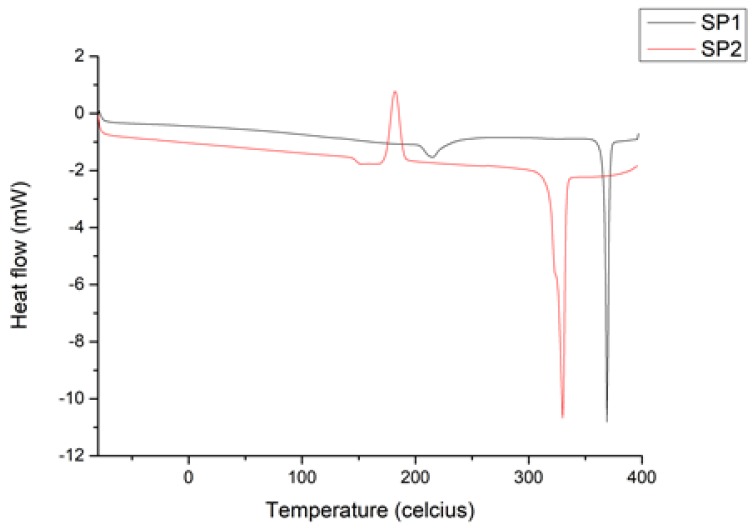
Differential scanning calorimetry (DSC) curves of diphenylacridine (SP1) and phenothiazine (SP2).

The material design not only improved the efficiencies, but also enhanced the intermolecular force, which is reflected in the differential scanning calorimetry (DSC) data (Table 1 and Figure 1). Both SP1 and SP2 showed high glass transition temperatures (*T*g) of 215 °C and 150 °C, respectively. Consequently, the thermal decomposition temperature of SP2 was 450 °C at 5% weight reduction. The commonly known hole transporting material NPB has a lower *T*g of 98 °C, which is not suitable for long-term device operations, but our materials can enhance morphological stabilities and support thin-film formation during the fabrication process [11]. The introduction of a spiro core intensified the thermal stabilities of SP1 and SP2, while the diphenylacridine core further built up the stabilities of SP1.

**Figure 2:**
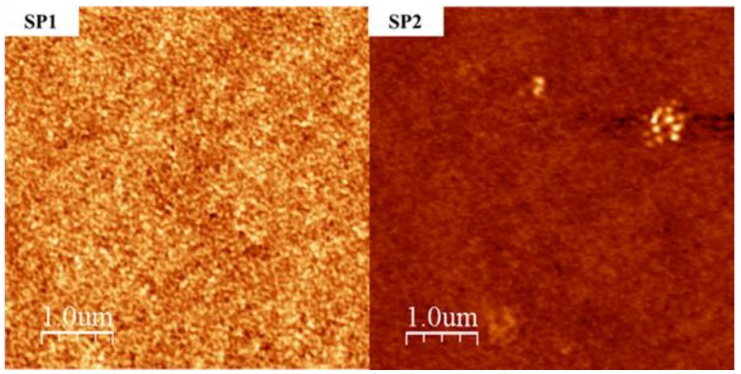
The scanning probe microscope (SPM) images of diphenylacridine (SP1) and phenothiazine (SP2).

The scanning probe microscope (SPM) is an ideal measurement for nanometric dimensional surface roughness and texture visualization (Figure 2).

**Figure 3:**
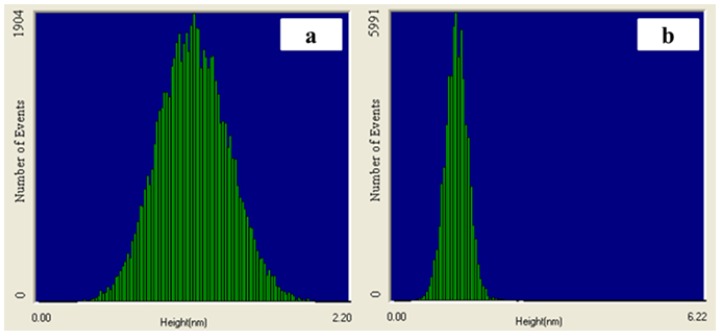
The height distribution of (**a**) diphenylacridine (SP1) and (**b**) phenothiazine (SP2).

We have measured the films with SP1 and SP2 HTMs to study the SPM images and the surface structure of the OLED film. The SP1-based device film had a root mean square roughness of 0.25 nm while the SP2-based device film had a roughness of 0.29 nm. The average roughness of the SP1 and SP2 films were 0.20 nm and 0.19 nm, respectively. The above values were calculated from the average value of the absolute distances of the surface points from the mean plane. The height distribution histograms helped to do the statistical analyses of the SPM data (Figure 3) [28,29].

The surface kurtosis value of the SP2-based film was 38.34, which was higher than that of the SP1-based film (3.02), and the higher values of the SP2-based film showed fewer high peaks and low valleys. Consequently, the skewness measurements of SP1 and SP2 were 0.10 and 3.36, respectively. Those values are in positive measurements, which can be applied to morphological applications. Moreover, SP2 revealed higher kurtosis and skewness values than SP1, which will help to improve the efficiency of the devices during long-term operations. We believe that SP2 will be more useful in thin-film applications when compare to SP1.

**Figure 4:**
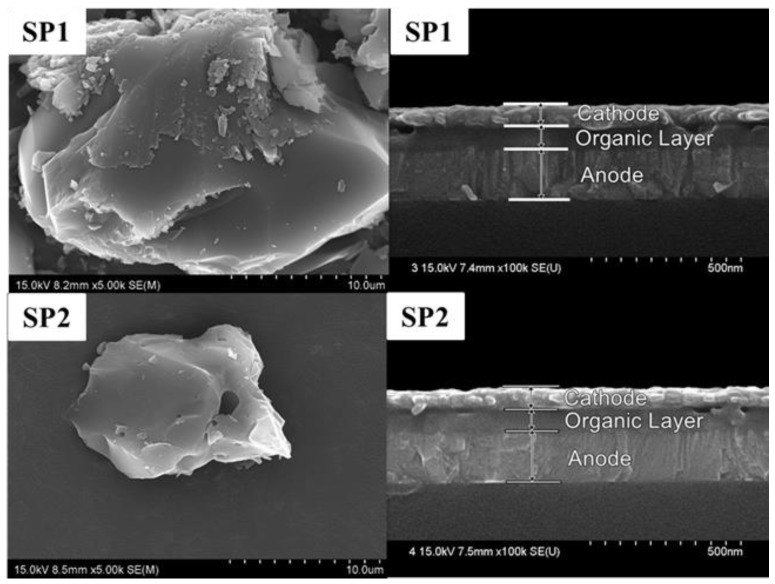
The scanning electron microscope (SEM) images of diphenylacridine (SP1) and phenothiazine (SP2) crystals and the cross-sectional views of the OLED devices.

The scanning electron microscope (SEM) images of the new materials, SP1 and SP2, and the cross-sectional views of the fabricated OLED devices are depicted in Figure 4. The cross-sectional SEM images of SP1 and SP2 exhibited well-defined multilayer structures with clear interfaces, which explain why our devices have good morphological characteristics. Additionally, we have provided the SEM images of the pure crystals of SP1 and SP2, which were taken before fabrication process.

### 2.3. Photophysical and Electrochemical Properties

**Figure 5:**
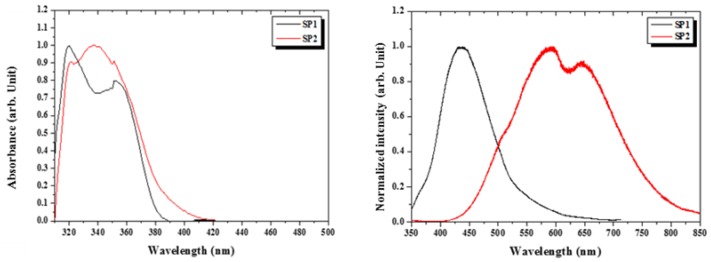
UV-visible absorption and photoluminescence (PL) spectra of diphenylacridine (SP1) and phenothiazine (SP2).

The UV-VIS absorption was measured at the film state, while the photoluminescence (PL) spectra of SP1 and SP2 were obtained at room temperature in a tetrahydrofuran (THF) solution (Figure 5). The details are summarized in Table 1. Both of the materials did not show any prominent absorption beyond 360 nm, which indicates that no absorption occurred in the visible region. The maximum peak of UV absorption was observed at 320 and 337 nm for SP1 and SP2, respectively. The above values might be ascribed to the transition of the molecular core and backbone. The onset absorption values for SP1 and SP2 were 395 and 422 nm, respectively, which helped to calculate the measurements of the energy band gaps of SP1 (3.14 eV) and SP2 (2.94 eV). The photoluminescence (PL) spectra of SP1 was 438 nm and SP2 showed a PL maximum of 593 nm. When we compare the PL maximum of both materials, SP2 showed red shifted wavelengths due to the phenothiazine group attached to the second and seventh positions of the spiro central core, and its PL emission was in the yellow region.

The electrochemical properties were analysed based on frontier molecular orbital energies (FMOs). The highest occupied molecular orbital (HOMO) energy levels of SP1 and SP2 were −5.86 eV and −5.57 eV, respectively (Table 1). SP2 revealed higher HOMO value than SP1, which may help to transport the holes from the anode with an effective charge hopping pathway. The lowest unoccupied molecular orbital (LUMO) energy values were −2.72 eV and −2.63 eV for SP1 and SP2, respectively, which were obtained by subtracting the HOMO values from the band gap energies.

**Figure 6:**
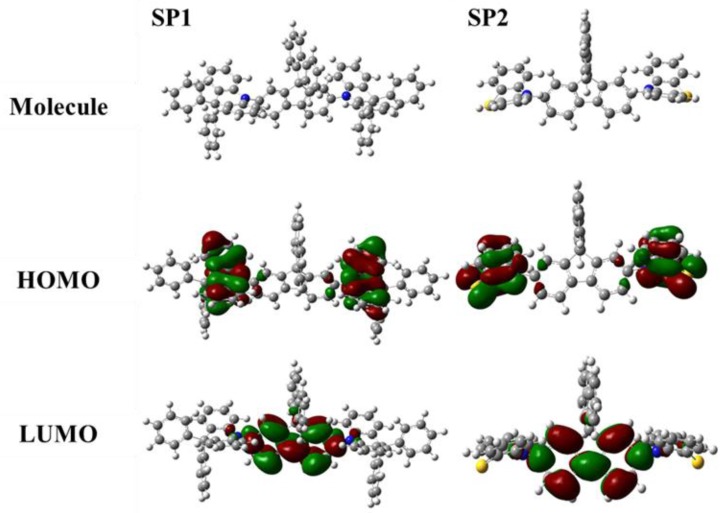
Molecular orbital simulation results of diphenylacridine (SP1) and phenothiazine (SP2).

The molecular orbital distribution of SP1 and SP2 were analysed by using density functional theory (DFT) calculation to evaluate the HOMO and LUMO distributions (Figure 6).

A suite of Gaussian 09 program was used along with the Time-Dependent Self-Consistent Field (TD-SCF) method B3LYP with a 6-31G basis set to calculate the molecular orbital distribution of the FMOs. We can clearly observe that the distribution occurred in a symmetrical way in both SP1 and SP2. Consequently, we could observe that there is a clear separation between the HOMO and LUMO. The HOMO values of SP1 and SP2 localized over acridine and phenothiazine moieties, respectively. The LUMO values of SP1 and SP2 were delocalized over fluorene units on the spiro core. Moreover, the simulation results exhibit that the hole transport properties of SP1 and SP2 are dominated by acridine and phenothiazine molecules.

### 2.4. Device Characteristics

**Figure 7:**
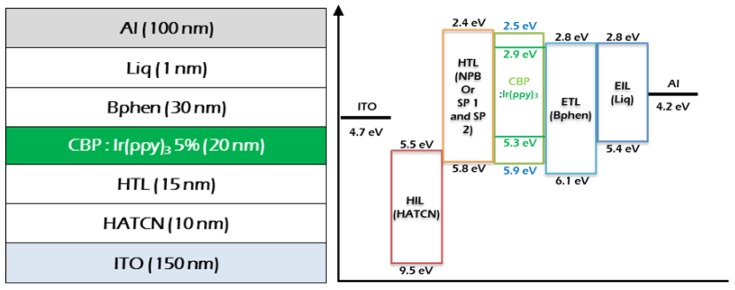
The OLED device structure and energy diagram of fabricated green devices.

The electroluminescence characteristics were investigated after fabricating green phosphorescent OLEDs with structures as follows: ITO (150 nm)/HATCN (10 nm)/HTL (15 nm)/CBP: 5 wt % Ir(ppy)_3_/Bphen (30 nm)/Liq (1 nm)/Al (100 nm). Indium tin oxide (ITO) was used as the anode because of its good conductivity, while Al was the cathode. 1,4,5,8,9,11-hexaazatriphenylenehexacarbonitrile (HATCN) and 8-quinolinolato lithium (Liq) were used for the hole injecting layer (HIL) and electron injecting layer (EIL). 4,4′-Bis(*N*-carbazolyl)-1,1′-biphenyl (CBP) was doped with an Ir(ppy)_3_ green emitter to form the emission layer (EML). The device structure and energy diagram are depicted in Figure 7.

**Figure 8:**
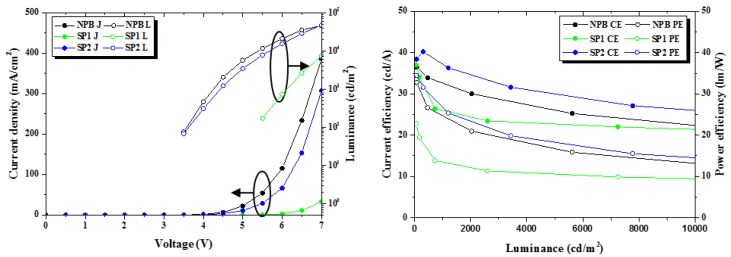
Current density-voltage-luminance (J-V-L) and luminescence current, power efficiency of the fabricated green PhOLED devices.

**Figure 9:**
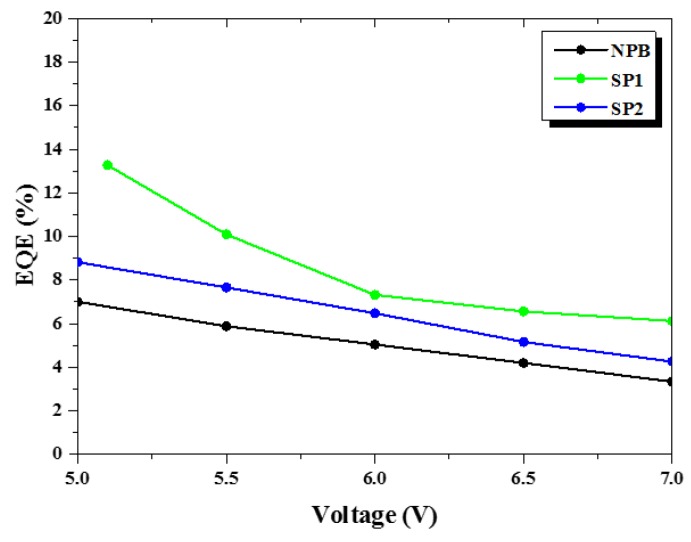
External quantum efficiencies of 4,4′-bis(*N*-phenyl-1-naphthylamino)biphenyl (NPB)-, diphenylacridine (SP1)-, and phenothiazine (SP2)-based devices.

To investigate SP1- and SP2-based device performances and efficiencies, a reference NPB-positioned device with a similar structure was constructed. The turn-on voltage of the SP2- based green device (3.5 V) was similar to that of the reference device (3.4 V). The current efficiency of SP2 at the turn-on voltage was 38.41 cd/A, while that of the reference device was 32.83 cd/A. The power efficiency of SP2 (34.47 lm/W) was much higher than that of SP1 (22.78 lm/W) because of its lower turn-on voltage. The abovementioned current density-voltage-luminance (J-V-L), current efficiency, and power efficiency data are illustrated in Figure 8 and summarized in Table 2. The external quantum efficiencies (EQE) of SP1 and SP2 were 13.27% and 13.43%, respectively, but the NPB-based reference device recorded a lower quantum efficiency of 11.45% (Figure 9). As a result, the OLED device fabricated with SP2 as the hole transporting material demonstrated better device properties than the reference and SP1-based devices.

**Figure 10:**
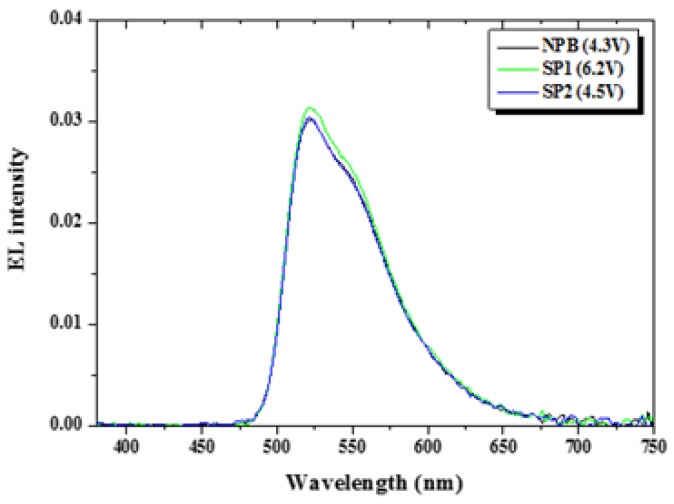
Electroluminescence (EL) spectra of 4,4′-bis(*N*-phenyl-1-naphthylamino)biphenyl (NPB), diphenylacridine (SP1), and phenothiazine (SP2) .

The electroluminescence (EL) spectra of all devices (Figure 10) showed a maximum peak at 524 nm, which originates from the green emission layer (EML), and we did not notice any other emissions from SP1 and SP2. The CIE color coordinate values were identical to each other with 0.33 for x and 0.62 for y.

## 3. Materials and Methods

### 3.1. General Procedures

All reagents and solvents were acquired from commercial suppliers and were used without further purification unless otherwise stated. The ^1^H- and ^13^C-NMR spectra were recorded by using a JNM-ECP FT-NMR spectrometer (JEOL, Peabody, MA, USA) operating at 500 MHz. The absorbance spectra were obtained from a S-4100 UV-visible spectrophotometer (SINCO, Seoul, Korea). Band gaps (Eg) were estimated from the onset of the absorbance spectra. The photoluminescence (PL) spectra were measured using a HR800 spectrofluorimeter (Horiba Jobin Yvon, Paris, France), and tetrahydrofuran (THF) was used as a solvent. The HOMO levels were calculated by AC-2 using a photoelectron spectrometer (RIKEN, Saitama, Japan). The LUMO levels were estimated from the obtained HOMO levels and band gaps by adding them together. The thermal gravimetric analysis was conducted on a DSC Q200 V24.9 Build 121 thermal analysis system (TA instruments, New Castle, DE, USA) at a heating rate of 10 °C/min. Mass analyses were carried out by using a Xevo TQ-S spectrometer (Waters, Milford, MA, USA). The scanning electron microscopic images were measured by a S-4800 SEM (Hitachi, Tokyo, Japan). Scanning probe microscopes (SPMs) were used to study the SPM images of the films (n-Tracer, Seoul, Korea). The molecular distributions were done by using a Gaussian 09 program (Wallingford, CT, USA) with DFT (density functional theory) and TD-SCF method B3LYP with a 6-31G basis set.

### 3.2. Synthetic Procedures

#### 3.2.1. 2,7-bis(9,9-Diphenylacridin-10(9*H*)-yl)-9,9′-spirobi[fluorene] (SP1)

A mixture of 2,7-dibromo-9,9′-spirobi[fluorene] (SP, 1 g, 1 equiv.), 9,9-diphenyl-9,10 dihydroacridine (1, 1.50 g, 2.1 equiv.), Pd(OAc)_2_ (0.05 g, 0.1 equiv.), *t*-Bu_3_P (10% in toluene, 0.15 mL, 0.03 equiv.), NaO*t*-Bu (0.81 g, 4 equiv.), anhydrous toluene (80 mL), and dry THF (20 mL) were added together and stirred under a nitrogen atmosphere for 8 h at 110 °C. After completion of the reaction, the crude mixture was extracted with dichloromethane and water. The organic layer was dried over anhydrous sodium sulphate and concentrated through a rotary evaporation process. The residue was purified by using silica column chromatography with a *n*-hexane:dichloromethane (4:1) solvent system to achieve SP1 as a white solid. Yield: 84%; ^1^H-NMR (CDCl_3_) δ 7.96–7.98 (d, *J* = 10 Hz, 2H), 7.74–7.76 (d, *J* = 10 Hz, 2H), 7.31–7.35 (t, *J* = 10 Hz, 2H), 7.15–7.20 (m, 16H), 6.90–6.98 (m, 18H), 6.79–6.81 (m, 6H), 6.59 (s, 2H), 6.26–6.28 (d, *J* = 10 Hz, 2H); ^13^C NMR (500 MHz, CDCl_3_) δ 151.91, 147.58, 146.57, 142.14, 141.90, 140.82, 140.75, 130.75, 130.40, 130.10, 129.42, 128.16, 128.08, 127.63, 127.57, 126.81, 126.26, 123.62, 122.30, 120.30, 120.15, 114.06, 56.68; GC-MS: 978.58 for C_75_H_50_N_2_ [M+H^+^].

#### 3.2.2. 2,7-di(10*H*-Phenothiazin-10-yl)-9,9′-spirobi[fluorene] (SP2)

A mixture of 2,7-dibromo-9,9′-spirobi[fluorene] (SP, 1 g, 1 equiv.), 10*H*-phenothiazine (2, 0.88 g, 2.1 equiv.), Pd(OAc)_2_ (0.05 g, 0.1 equiv.), *t*-Bu_3_P (10% in toluene, 0.15 mL, 0.03 equiv.), NaO*t*-Bu (0.81 g, 4 equiv.), and anhydrous toluene (100 mL) were added together and stirred under a nitrogen atmosphere for 6 hours at 110 °C. After completion of the reaction, the crude mixture was extracted with dichloromethane three times. The organic layer was dried over anhydrous sodium sulphate and concentrated through a rotary evaporation process. The residue was purified by using chloroform and *n*-hexane solvents through a recrystallization process to achieve SP2 as a yellow solid. Yield: 92%; ^1^H-NMR (500 MHz, CDCl_3_) δ 8.12–8.14 (d, *J* = 10 Hz, 2H), 7.75–7.77 (d, *J* = 10 Hz, 2H), 7.44–7.46 (d, *J* = 10 Hz, 2H), 7.31–7.34 (t, *J* = 10 Hz, 4H), 7.11–7.14 (t, *J* = 10 Hz, 4H), 6.87–6.94 (m, 10H), 6.81 (s, 2H), 6.08–6.10 (d, 4H); ^13^C NMR (500 MHz, CDCl_3_) δ 152.66, 147.34, 141.93, 140.86, 128.34, 128.12, 126.83, 126.68, 123.60, 122.21, 120.45, 115.70, 66.18; GC-MS: 710.50 for C_49_H_30_N_2_S_2_ [M + H^+^].

### 3.3. OLED Fabrication and Characterization

Green phosphorescent OLED devices were constructed to study our designed materials’ efficiencies and performances. The device substrate was made by using an ITO with a thickness of 150 nm. Then, the substrate was subjected to ultra-sonication with isopropyl alcohol and deionized water, followed by ultraviolet and ozone treatment. Further device fabrication continued as follow: 1,4,5,8,9,11-hexaazatriphenylenehexacarbonitrile (HATCN) was used as the hole injecting layer (HIL), 4,4′-bis(*N*-phenyl-1-naphthylamino)biphenyl (NPB) was the reference hole transporting material (HTM), Tris[2-phenylpyridinato-C^2^,*N*]iridium(III) (Ir(ppy)_3_) was used for green dopant at the emission layer doping with 4,4′-Bis(*N*-carbazolyl)-1,1′-biphenyl (CBP) as the host material, bathophenanthroline (Bphen) was used as the electron transporting material, 8-quinolinolato lithium (Liq) was used as the electron injecting layer (EIL), and aluminium (Al) was the cathode. All organic layers were deposited by a thermal evaporating system under 5 × 10^−7^ torr pressure (Sunicel plus, Seoul, Republic of Korea). Finally, the deposited devices were encapsulated with glass. Current density-voltage-luminance (J-V-L) efficiencies were measured by an OLED J-V-L test system (Polarmix M6100, Suwon, Korea). The electroluminescence (EL) spectra analysis was carried out by using a spectroradiometer (Konica Minolta CS-2000, Tokyo, Japan).

## 4. Conclusions

In conclusion, two novel hole transporting materials, SP1 and SP2, were designed and synthesized. The synthesized materials were applied on green phosphorescent OLEDs as hole transporting materials, which exhibited high glass transition temperatures of over 150 °C. SP2 exhibited excellent current (38.41 cd/A) and power efficiencies (34.47 lm/W), while SP1 had a better current efficiency of 36.98 cd/A, but a lower power efficiency because of its higher turn-on voltage of 5.1 V. The external quantum efficiency (EQE) of SP2 was at 13.43%, which is 2% higher than that of the reference NPB-based device. This study brought an outstanding perception of how a spiro compound with different modifications at the second and seventh position could enhance device efficiencies and hole transporting capability with thermal stabilities. Our SP2 hole transporting material will be a promising contender for future green phosphorescent OLED applications and will helpful to develop further spiro-based OLED materials with higher efficiencies.

## Figures and Tables

**Table 1:** Thermal and physical properties of diphenylacridine (SP1) and phenothiazine (SP2). Hole Transporting Materials (HTM), Photoluminescence Spectra (PL).

HTMs	*T*_g_^a^ (°C)	UV-VIS ^b^ (nm)	PL Max (nm)	HOMO (eV)	LUMO (eV)	*E_g_*^c^ (eV)	*E_T_*^d^ (eV)
SP1	215	395	438	5.86	2.72	3.14	2.83
SP2	150	422	593	5.57	2.63	2.94	2.09

^a^ Glass transition temperature, ^b^ onset UV absorption, ^c^ band gap energy, ^d^ triplet energy.

**Table 2:** Device characteristics of diphenylacridine (SP1) and phenothiazine (SP2). 4,4′-bis(*N*-phenyl-1-naphthylamino)biphenyl (NPB).

Device Properties	NPB	SP1	SP2
Turn-on voltage (V)	3.4 ^a^	5.1 ^a^	3.5 ^a^
Driving voltage (V)	4.3 ^b^	6.2 ^b^	4.5 ^b^
Current (mA)	0.16 ^b^	0.20 ^b^	0.13 ^b^
Current efficiency (cd/A)	32.83 29.52 ^b^	36.98 24.88 ^b^	38.41 36.33 ^b^
Power efficiency (lm/W)	30.33	22.78	34.47
EQE	11.45% 8.19% ^b^	13.27% 6.87% ^b^	13.43% 10.27% ^b^
CIE (x,y)	(0.33, 0.62)	(0.33, 0.63)	(0.33, 0.62)

^a^ Measured at 1 cd/m^2^, ^b^ Measured at 1200 cd/m^2^.
